# Probing artificial intelligence in neurosurgical training: ChatGPT takes a neurosurgical residents written exam

**DOI:** 10.1016/j.bas.2023.102715

**Published:** 2023-11-29

**Authors:** A. Bartoli, A.T. May, A. Al-Awadhi, K. Schaller

**Affiliations:** a Department of Clinical Neurosciences, Division of Neurosurgery, Geneva University Medical Center, Geneva, Switzerland; b Switzerland & Faculty of Medicine, University of Geneva, Switzerland

**Keywords:** Artificial intelligence, ChatGPT, Neurosurgical education, Written exam, Residents

## Abstract

**Introduction:**

Artificial Intelligence tools are being introduced in almost every field of human life, including medical sciences and medical education, among scepticism and enthusiasm.

**Research question:**

to assess how a generative language tool (Generative Pretrained Transformer 3.5, ChatGPT) performs at both generating questions and answering a neurosurgical residents’ written exam. Namely, to assess how ChatGPT generates questions, how it answers human-generated questions, how residents answer AI-generated questions and how AI answers its self-generated question.

**Materials and methods:**

50 questions were included in the written exam, 46 questions were generated by humans (senior staff members) and 4 were generated by ChatGPT. 11 participants took the exam (ChatGPT and 10 residents). Questions were both open-ended and multiple-choice.

8 questions were not submitted to ChatGPT since they contained images or schematic drawings to interpret.

**Results:**

formulating requests to ChatGPT required an iterative process to precise both questions and answers. Chat GPT scored among the lowest ranks (9/11) among all the participants). There was no difference in response rate for residents’ between human-generated vs AI-generated questions that could have been attributed to less clarity of the question. ChatGPT answered correctly to all its self-generated questions.

**Discussion and conclusions:**

AI is a promising and powerful tool for medical education and for specific medical purposes, which need to be further determined. To request AI to generate logical and sound questions, that request must be formulated as precise as possible, framing the content, the type of question and its correct answers.

## Highlights

• This study results from our first experience at integrating an artificial intelligence tool in a human-based process, such as elaborating and taking a written exam for neurosurgical trainees. We integrated ChatGPT 3.5 at both generating questions as well as participating to the written exam along with other 10 participants (humans).
• The written exam is taken every from our trainees in order to train for the EANS Part I exam, which is compulsory to access the Swiss final federal board exam for the completion of training. It consisted of 50 questions and 4/50 were generated by ChatGPT. Considering it being our first experience we kept the number relatively low. On the other side, ChatGPT was submitted with 42 questions (8 questions were excluded since they contained mainly images to interpret).
• The interaction with the AI, the formulation of questions as well as the answers generated, proved to be an exercise of precision of language and appropriate content framing.
• We do believe that this study is appropriate for Brain & Spine as an official journal of the European Association of Neurosurgical Societies, actively involved in neurosurgical education and can benefit readers on a compelling topic like artificial intelligence in neurosurgery.
• The study reported in this manuscript is original and it has not been previously published in whole or in part or under submission elsewhere for review.

## 1 Introduction

Artificial intelligence tools create shockwaves in all fields of daily life, amongst blind enthusiasm, hyperbolic headlines, and apocalyptic fear by some (Hutson, 2023; Lewis), like most newly introduced technologies in the past. Medical science has not been spared and ChatGPT has been tested in various medical fields (Hamet and Tremblay, 2017; Kulkarni et al., 2020) as well as science writing in general (Tools such as ChatGPT threaten, 2023) and medical education (Gilson et al., 2023).

We sought to test ChatGPT tool in neurosurgical education: more precisely we integrated ChatGPT tool in our annual neurosurgical residents’ exam, for both generating questions and taking the exam itself.

As part of their training, neurosurgical residents at Geneva University Hospitals undergo an annual written exam to test their knowledge and to train for the European Association of Neurosurgical Societies written exam (Part I), which is a compulsory step before taking the Swiss federal board exam (oral) and obtain the board certification (certificate of completion of training).

### 1.1 Our aim was twofold

1. To mix ChatGPT-generated questions with questions generated by senior staff, and to assess if there was a difference in the residents' response rate (i.e. due to lesser understandability of ChatGPT-generated vs. human-made questions).
2. The exam contained man-made questions requiring free text answers. By letting ChatGPT participate in the exam, we wanted to assess how ChatGPT would perform as compared to neurosurgical residents (PGY1-4) in both parts of the exam – multiple choice questions and open questions, respectively.

## 2 Methods

ChatGPT (Generative Pretrained Transformer 3.5) was included for both generating questions and taking the written exam.

The written exam consisted of 50 questions, either multiple choices (with both single-select and multiple-select answers) or open-ended questions (with predefined keywords answers to confer points).

4/50 questions were generated by ChatGPT by formulating different requests. The other 46 questions were formulated by junior and senior local attending neurosurgeons (all completed formal neurosurgical training) and supervised by the first author. Expected correct answers were given by each attending neurosurgeon and a separate grid with all the right answers was generated. 7/46 questions included pictures (i.e. MRI scans, CT scans, surgical technique description, surgical anatomy) and 1 question was about a specific institutional protocol for surgical site infection after spinal instrumentation.

11 participants took the exam (10 residents and ChatGPT): 10 residents took the entire exam while ChatGPT took 42 questions (questions including pictures and the institutional protocol were excluded). The 11 participants had 60 min to complete the exam.

Questions for ChatGPT were copy-pasted in the chat-box and answers were recorded and assessed according to predefined criteria.

We therefore proceeded to assess the following:

1. How ChatGPT generated questions for a written exam for neurosurgical residents
2. How ChatGPT answered a written exam for neurosurgical residents and how it ranks among 1st to 4th year post-graduate trainees in neurosurgery
3. How residents answered and scored at ChatGPT generated questions
4. How ChatGPT answered its self-generated questions

## 3 Results

Overall, ranking and scores of all participants were as follows:

**Table:** 

RANKING	RESIDENT	SCORE
1	A	39.25
2	B	35.45
3	C	29
4	D	26.9
5	E	26.35
**6**	**ChatGPT**	**25.4**
7	F	24.45
8	G	24
9	H	22.2
10	I	19.2
11	J	15.25

ChatGPT scored 21.4 (25.5/50 weighted over 42 questions) and ranked 6th out of 11 candidates.

There was no difference in residents’ response rate when comparing human-generated questions to ChatGPT generated questions. One question generated by ChatGPT (No.47) was consistently failed by all the residents.

### 3.1 Generating questions with chat GPT

Questions were generated by asking ChatGPT the following:

Question 47.

**Table:** 

Request to AI chat-box	*“Please generate one question and multiple choices answers and the right answer for a neurosurgical residents exam”*
Generated answer	**A 65 year old male patient presents with progressive weakness and numbness in the right lower extremity. Neurological examination reveals hyperreflexia and positive Babinski sign on the right side. An MRI scan shows a large intradural extramedullary tumor located at the level of the thoracic spinal cord. What is the most likely diagnosis for this patient?** A) Meningioma B) Glioblastoma C) Schwannoma D) Ependymoma E) Medulloblastoma Answer: C) Schwannoma

Question 48.

**Table:** 

Request to AI chat-box	*“Please generate one question and multiple choices answer for a neurosurgical residents exam”*
Generated answer	**A 45 year old patient presents with severe headaches, visual disturbances, and bitemporal hemianopia. Magnetic resonance imaging (MRI) reveals a large pituitary tumor compressing the optic chiasm. What's the most appropriate treatment for this patient?** A) Craniotomy and microsurgical resection of the tumor B) Gamma Knife radiosurgery C) Transsphenoidal endoscopic resection of the tumor D) Medical management with dopamine agonists E) Observation and follow-up with serial imaging (Note: The answer choices are based on common treatment options for pituitary tumours, and the most appropriate choice may vary depending on the specific clinical scenario.)

Question 49.

**Table:** 

Request to AI chat-box	*“Please generate one open question with the right answers for a neurosurgical residents exam”*
Generated answer	**Describe the clinical features and diagnostic workup of a patient presenting with symptoms of hydrocephalus. What are the appropriate treatment options for this condition?** Clinical features: Diagnostic workup: Treatment options: Possible Answers: Clinical Features: Symptoms of hydrocephalus may include headache, nausea, vomiting, altered mental status, gait disturbances, urinary incontinence, and visual changes. Physical examination may reveal signs of increased intracranial pressure such as papilledema and cranial nerve abnormalities. Diagnostic Workup: The diagnostic workup for hydrocephalus typically involves brain imaging, such as a computed tomography (CT) scan or magnetic resonance imaging (MRI) of the brain, to evaluate the ventricular size and identify any underlying causes. Additionally, lumbar puncture may be performed to measure the opening pressure and analyse the cerebrospinal fluid (CSF). Treatment Options: The treatment options for hydrocephalus depend on the underlying cause and the severity of symptoms. They may include: • Shunt Placement: The most common treatment for hydrocephalus is the surgical placement of a ventriculoperitoneal (VP) shunt. This involves creating a tunnel from the ventricles of the brain to the peritoneal cavity, allowing excess CSF to drain and relieve pressure. • Endoscopic Third Ventriculostomy: In selected cases, an endoscopic third ventriculostomy (ETV) may be performed instead of shunt placement. This procedure creates an opening in the floor of the third ventricle, allowing CSF to flow out of the ventricular system and into the subarachnoid space. • Treatment of Underlying Cause: In some cases, hydrocephalus is secondary to an underlying condition, such as a tumour or infection. Treating the underlying cause may alleviate the hydrocephalus, either through surgical resection, medication, or antimicrobial therapy. It is important to note that the appropriate treatment approach should be determined based on the individual patient's condition, the cause of hydrocephalus, and the clinical judgment of the neurosurgeon.

Question 50.

**Table:** 

Request to AI chat-box	*“Please generate one multiple choice question and its right answer of neurosurgical anatomy for residents”*
Generated answer	**Which structure serves as a landmark to identify the lateral ventricle during neurosurgical procedure?** A) Cingulate gyrus B) Corpus callosum C) Thalamus D) Hippocampus Right Answer: B) Corpus callosum

It is interesting to note how questions are formulated depending on how precise the human request is formulated.

Namely, in question 48 the request is formulated without asking for the right answer(s) and the tool generates a question were all the answers are correct indeed, and giving a disclaimer note. In such a question 1 point was given to all participants.

In question 47 and 50 the request is explicitly formulated by asking not only the question itself but also one and one only right answer, to which the tool replies precisely.

In question 49 the request is formulated so to generate an open-ended question. The answer provided by ChatGPT about clinical features, diagnostic workup and treatment options for hydrocephalus provided the keywords used to assess how the other participants (residents) answered. Its formulation though remains somewhat evasive since it does not specify the type of hydrocephalus (e.g. acute/subacute/chronic, normal pressure, obstructive etc.) and its nuances about clinical scenario and treatment options.

#### 3.1.1 ChatGPT taking a neurosurgical resident exam

42 questions out of 50 were submitted to the ChatGPT chatbox. 8 questions were exlcuded since they contained images to interpret (radiological, schematic surgical technique drawings or surgical anatomy views) and/or local-institutional clinical protocol that the tool could have not reasonably known. The exam that was taken by ChatGPT along with its answers is shown in the attached document (ChatGPT Exam, Supplementary Material).

For instance, question 5 posited initially as follows:

**Table:** 

Which is the only pelvic parameter that stays constant all the life long? Pelvic Tilt Sacral Slope Pelvic Incidence Lumbar Lordosis Sagittal Vertical Axis

To which AI provided a misled answer, related to a gynaecological-obstetrics parameter:

**Table:** 

The only pelvic parameter that remains constant throughout a person's life is the Pelvic Inlet. The Pelvic Inlet, also known as the Superior Pelvic Aperture, is the opening at the upper part of the pelvis. It is bordered by the pelvic bones and is the entrance into the pelvic cavity. The size and shape of the Pelvic Inlet are established during early development and remain relatively stable throughout adulthood. It serves as a fixed structure that defines the dimensions and limits of the pelvic cavity. The size of the Pelvic Inlet is important for childbirth as it determines the passage through which the baby's head must pass during delivery.

None of the other 10 participants was misled by the way the question was formulated.

In order for ChatGPT to frame its answer in alignment with neurosurgical content, we reformulated the question as follows:

**Table:** 

Which is the only spino-pelvic parameter that stays constant all the life long? Pelvic Tilt Sacral Slope Pelvic Incidence Lumbar Lordosis Sagittal Vertical Axis

Overall, ChatGPT ranked in the middle scores out of 11 participants.

#### 3.1.2 Neurosurgical residents answering ChatGPT-generated questions

Question 47 was consistently given an incorrect answer from all residents, who mostly answered “meningioma” whereas the expected AI-generated answer was “schwannoma”. This does not seem related to the understability of the question per se but to a genuine lack of knowledge.

Question 48 included potentially all correct answers, since “most appropriate treatment for a large pituitary tumor” encompasses all the given options depending on the clinical scenario: all residents received one point regardless of the chosen answer. Question 49 was correctly answered by most residents despite its broad content. Question 50, which included a more specific anatomical concept, was answered correctly by 6/10.

#### 3.1.3 .ChatGPT answering self-generated questions

Not surprisingly, chat GPT answered correctly to the four self-generated questions, No. 47 to 50.

## 4 Discussion

Generative language models (including ChatGPT) work in complex ways that are also proprietary, but the basic principles are public and accessible to everyone (Stollnitz, 2023).

Artificial Intelligence has been already largely tested in various fields of medicine (Hamet and Tremblay, 2017; Kulkarni et al., 2020) as well as in decision-making processes in neurooncology (Haemmerli et al., 2023) and medical writing (Kitamura, 2023) in general, raising questions about transparence and falsehood in science (Tools such as ChatGPT threaten, 2023).

A recent study showed that ChatGPT achieves the equivalent of a passing score for a third year medical student on the United States Medical Licensing Examination: authors also highlighted “ChatGPT's capacity to provide logic and informational context across the majority of answers” (Gilson et al., 2023).

In the present experience, generating questions with ChatGPT proved to be somehow less intuitive than expected. The initial human request that was submitted to the AI chatbox was as follows:

“*please generate three questions for neurosurgical residents exam*”

And these were the suggested AI-generated questions:

1. Describe the steps involved in performing a craniotomy for tumor resection, including preoperative planning, positioning, and intraoperative techniques to minimize complications.
2. Discuss the management of a patient presenting with acute traumatic brain injury, including initial stabilization, imaging modalities for diagnosis, and surgical interventions for intracranial hemorrhage or raised intracranial pressure.
3. Explain the indications, surgical approaches, and potential complications associated with deep brain stimulation (DBS) surgery for the treatment of movement disorders such as Parkinson's disease or essential tremor. Additionally, outline the postoperative management and long-term outcomes of DBS patients.

These questions were eventually not included in the final exam because, despite being well-formulated and consistent with the submitted human request, provided a much broader content and no criteria to define a correct answer. At this stage, it seemed like AI did not “intend” or “think” to probe someone's specific knowledge on a topic when generating a question, like humans do.

The 4 AI-generated questions were eventually formulated by redefining in a more precise way the human request, as shown above. This included requesting specifically the type of question (e.g. multiple choice) as well as the right answer(s) (e.g. question 47). In question 48, we did not request to generate the right answer to a multiple-choice question and the tool generated multiple answers that were potentially all correct indeed, depending on the specific clinical scenario.

When requesting ChatGPT to generate an open question with its right open answer (question 49), the results were again quite generic in content and gave room to a lot of possible interpretation and questioning from residents. A few residents commented indeed on this question: “*does it refer to chronic or acute hydrocephalus*?” “*what is the precise clinical setting*?“. Moreover, it was probably not an ideal question for a 50 questions-exam to be taken in 60 min since it involved an extensive text-writing.

On the other hand, question 50 provides a good example of a precise enough question generated by the AI since its original request specifies the content (neurosurgical anatomy), the type of question (multiple choice) and one and only one right answer.

Generating questions “de novo” for a neurosurgical residents exam can be time consuming for humans, since it requires a certain amount of knowledge, creativity, thinking, precision and verification of contents: the speed of processing a vast amount of information that AI has been trained on is with no objection incomparable to human brain, and while AI can pick hundreds of different neurosurgical topics in no time, it still requires precision and verification from humans.

Although very useful for quickly generating neurosurgical questions with little effort, a doublecheck by a neurosurgeon was necessary to filter out the right questions.

With hindsight, human-generated questions were possibly not precise enough as for their request and despite an overall correct answer from ChatGPT, they were not matching the human-expected answer. For example in question 1 “*What are the different types of white matter tracts of the brain*” the human-expected answer was “*projection, associative, commissural fibers”* whereas ChatGPT gave a much broader answer that was correct per se, yet not matching the expected answer. It is hard to unequivocally interpret the final score of ChatGPT to this test. While its knowledge is based on highly sophisticated statistical processes on texts that are regularly updated, it is also very likely that how human language generates and formulates questions is not always as clear as in the intention of the questioner, reminding us how our own language can be flawed and the necessity of being precise when we speak. Moreover, we must take into consideration that a certain bias may exist towards human-made questions, regardless of how well these are formulated. Indeed, the attending neurosurgeons that have formulated most questions have had a similar neurosurgical training to the residents whose training they also contribute to, and they tend to share the same language and semantics. Therefore, in the context of comparable training experiences, a degree of expectation for questions, answers and the intention behind them could have an influence. Residents may thus have more tolerance towards an unclear human-generated question compared to an unclear AI-generated question, as the intention behind the human-generated question may be more readily decrypted by a human. Similarly, the AI tool may have more difficulty answering unclear human-generated questions as it may not completely understand the intention hidden behind.

Eminent linguist and thinker N. Chomsky states: “The human mind is not, like ChatGPT and its ilk, a lumbering statistical engine for pattern matching, gorging of terabytes of data and extrapolating the most likely conversational response or most probable answer to a scientific question. On the contrary, the human mind is a surprisingly efficient and even elegant system that operates with small amounts of information; it seeks not to infer brute correlations among data points but to create explanations. (…) Machine learning systems can learn both that the earth is flat and that the earth is round. They trade merely in probabilities that change over time.” (Chomsky et al., 2023).

Powerful AI tools such as generative language models definitely have a potential in the medical field and can help standardize and bring excellent to neurosurgical care worldwide. Different ways to exploit AI for medical education and exams are imaginable: i.e. questions may be posed by a human, an answer is generated by AI, and a critical appraisal is then elaborated by the human, based on its own logical thinking, knowledge, and personal experience and creativity. Furthermore, besides education purposes, these AI tools could help query information in ever growing medical databases and knowledge, process that information and help come up with simplified answers, thus helping humans reduce the time input and scope of their research. These examples support the utility of AI tools to increase the efficiency of human labour, as a complement to humans rather than as a replacement.

The next logical experience in this field would be to compare results and rankings of neurosurgical residents when taking a fully AI-generated exam vs a fully human-generated exam.

## 5 Conclusion

It is evident that AI is about to become a powerful tool for medical education and for specific medical purposes, which need to be further determined. To request AI to generate logical and sound questions, that request must be formulated as precisely as possible, framing the content, the type of question and its correct answers.

## Disclosure of funding

None

On behalf of all the authors, thank you for your consideration.

## Disclosure

The authors have no conflicts of interest to declare. All co-authors have seen and agree with the contents of the manuscript and there is no financial/non-financial interest to report.

## Declaration of competing interest

None.
