# Supplementary material for: Probing artificial intelligence in neurosurgical training: ChatGPT takes a neurosurgical residents written exam
Source: Brain Spine. 2023 Nov 29;4:102715. doi: 10.1016/j.bas.2023.102715 (PMC10753430; doi:10.1016/j.bas.2023.102715)
Supplement: Multimedia component 1 [file mmc1.docx]

1. **What are the different types of white matter tracts of the brain**

ChatGPT

White matter tracts in the brain are bundles of nerve fibers (axons) that connect different regions of the brain and enable communication between them. There are several major white matter tracts in the brain, and here are some of the most prominent ones:

1. Corpus Callosum: This is the largest white matter tract in the brain and connects the two cerebral hemispheres. It facilitates communication and coordination between the left and right sides of the brain.
2. Anterior Commissure: This tract connects the anterior regions of the cerebral hemispheres, primarily the frontal lobes. It plays a role in olfactory (smell) and visual processing.
3. Posterior Commissure: This small tract connects the posterior regions of the cerebral hemispheres and is involved in visual reflexes and pupillary control.
4. Internal Capsule: The internal capsule is a dense bundle of white matter fibers that passes between the cerebral cortex and the brainstem. It consists of anterior, posterior, and sublenticular parts. The internal capsule is a major pathway for motor and sensory fibers traveling to and from the cerebral cortex.
5. Corona Radiata: This fan-shaped white matter tract extends from the internal capsule and spreads out across the cerebral cortex. It contains ascending and descending fibers that connect the cortex to other regions of the brain.
6. Cingulum: The cingulum is a bundle of fibers that encircles the corpus callosum. It connects various limbic structures, such as the hippocampus and cingulate gyrus, and is involved in emotion, memory, and attention.
7. Arcuate Fasciculus: This tract connects Broca's area, responsible for language production, with Wernicke's area, responsible for language comprehension. It plays a crucial role in language processing and communication.
8. Superior Longitudinal Fasciculus: Also known as the superior longitudinal bundle, this tract connects the frontal, parietal, and occipital lobes. It is involved in higher cognitive functions such as attention, working memory, and spatial processing.
9. Uncinate Fasciculus: The uncinate fasciculus connects the frontal lobe, particularly the orbitofrontal cortex, with the temporal lobe, including the amygdala and hippocampus. It is involved in emotional processing and memory.

These are just a few examples of the white matter tracts in the brain. The brain's white matter is a complex network of interconnected fibers, and ongoing research continues to unveil more details about its structure and function.

1. **Name 4 projection fibers**

ChatGPT

Projection fibers are a type of white matter tract that connects different regions of the brain to distant areas. They are responsible for transmitting information between the cerebral cortex and other structures. Here are four examples of projection fibers:

1. Corticospinal Tract: The corticospinal tract, also known as the pyramidal tract, is a major projection fiber that originates in the motor cortex of the cerebral cortex and descends to the spinal cord. It is involved in the voluntary control of movement.
2. Corticobulbar Tract: The corticobulbar tract is another projection fiber originating from the motor cortex. It projects to the brainstem, specifically the cranial nerve nuclei, which control the muscles of the face, head, and neck. This tract is responsible for voluntary movements involved in facial expressions, chewing, swallowing, and speaking.
3. Thalamocortical Radiations: These projection fibers connect the thalamus, a central relay station for sensory information, with the cerebral cortex. They transmit sensory signals from various sensory modalities (e.g., vision, hearing, touch) to the appropriate cortical areas for further processing.
4. Striatum (Basal Ganglia) Pathways: The basal ganglia, a group of subcortical nuclei involved in motor control and other functions, has multiple projection fibers connecting it with the cerebral cortex. These pathways, including the direct and indirect pathways, play a crucial role in regulating motor movements and influencing cognitive functions.

These are just a few examples of the projection fibers in the brain. The brain's white matter is a complex network with numerous tracts facilitating communication between different brain regions.

1. **In deep brain stimulation, what nucleus is usually targeted for Essential Tremor (ET) and which one for Parkinson Disease (PD)?**

ChatGPT

In deep brain stimulation (DBS), a surgical procedure is performed to implant electrodes in specific brain regions to treat certain neurological conditions. The choice of the target nucleus depends on the particular condition being treated.

For Essential Tremor (ET), the thalamic nucleus that is typically targeted is the Ventral Intermediate Nucleus (VIM) of the thalamus. The VIM is involved in motor control, and its stimulation through DBS can help reduce the tremors associated with Essential Tremor.

For Parkinson's Disease (PD), the two most common target nuclei for DBS are the Subthalamic Nucleus (STN) and the Globus Pallidus Internus (GPi). The selection between these two targets depends on various factors, including the individual patient's symptoms and response to medication. Stimulation of these nuclei can help alleviate the motor symptoms of Parkinson's Disease, such as tremors, rigidity, and bradykinesia (slowness of movement).

It's important to note that the specific choice of target nucleus may vary depending on the patient's condition and the judgment of the neurologist and neurosurgeon involved in the DBS procedure.

1. **Define the lamination of the corticospinal tract**

ChatGPT

The lamination of the corticospinal tract refers to the organization and layering of the nerve fibers within this tract as they descend from the cerebral cortex to the spinal cord. The corticospinal tract is a major pathway responsible for voluntary motor control, transmitting signals from the motor cortex to the spinal cord, which then innervates muscles throughout the body.

The lamination of the corticospinal tract consists of two main divisions: the lateral corticospinal tract and the anterior corticospinal tract.

1. Lateral Corticospinal Tract: The majority of the corticospinal fibers form the lateral corticospinal tract. These fibers originate from the primary motor cortex (precentral gyrus) and the premotor cortex. They decussate (cross over) from one side of the brain to the opposite side in the medulla, specifically in an area called the pyramidal decussation. Once they cross, they continue down the contralateral (opposite) side of the spinal cord. In the spinal cord, these fibers primarily synapse with interneurons and motor neurons in the lateral gray matter, which then transmit motor signals to muscles involved in fine motor control, especially distal muscles of the limbs.
2. Anterior Corticospinal Tract: A smaller portion of corticospinal fibers forms the anterior corticospinal tract. Unlike the lateral corticospinal tract, these fibers do not decussate at the level of the pyramidal decussation. Instead, they continue down the ipsilateral (same) side of the spinal cord. Upon reaching the spinal cord, these fibers either decussate or synapse directly with interneurons and motor neurons in the anterior gray matter. The anterior corticospinal tract is involved in controlling bilateral and axial (trunk) muscles.

It's important to note that the corticospinal tract does not have a distinct anatomical layering within the spinal cord, as the term "lamination" typically refers to layering within the cerebral cortex. The description above highlights the division of the corticospinal tract into lateral and anterior components based on their pathway and decussation patterns.

1. **Which is the only spino-pelvic parameter that stay constant all the life long?**

Pelvic Tilt

Sacral Slope

Pelvic Incidence

Lumbar Lordosis

Sagittal Vertical Axis

ChatGPT

The only spino-pelvic parameter that remains constant throughout a person's life is the Sacral Slope. The Sacral Slope is a measure of the angle between the horizontal plane and the sacral plate, which is the base of the sacrum bone located at the lower part of the spine.

Unlike other spino-pelvic parameters such as pelvic tilt, pelvic incidence, or lumbar lordosis, which can vary due to age, posture, or pathological conditions, the Sacral Slope remains relatively stable and does not significantly change over time. It is determined by the shape and orientation of the sacrum and does not undergo significant alterations during growth or adulthood.

The Sacral Slope is an important parameter in assessing spinal alignment and balance. It is often used in conjunction with other spino-pelvic parameters to evaluate conditions such as spinal deformities, postural imbalances, or spine-related disorders.

1. **How many degrees of lordosis we may obtain with a Smith-Petersen osteotomy**

5-10°

30°

2°

40°

10-15°

ChatGPT

A Smith-Petersen osteotomy is a surgical procedure performed on the spine to correct certain spinal conditions, such as excessive lordosis or kyphosis. It involves removing a wedge-shaped section of bone from the vertebrae to change the alignment and curvature of the spine.

The degree of lordosis that can be obtained with a Smith-Petersen osteotomy can vary depending on several factors, including the specific technique used, the number of levels involved in the procedure, and the patient's individual anatomy and condition.

In general, a Smith-Petersen osteotomy can achieve a correction of lordosis ranging from a few degrees to around 20 degrees. However, it's important to note that the actual degree of lordotic correction achieved can be influenced by various factors and should be determined by the surgeon during the surgical planning and evaluation process.

It's crucial for individuals considering a Smith-Petersen osteotomy or any spinal surgery to consult with a qualified spine surgeon who can provide a thorough evaluation, discuss the potential outcomes, and determine the most appropriate approach for their specific case.

1. **How do you classify this occipital condyle fracture? And which is the involved ligament? (NOT SUBMITTED TO CHAT GPT)**


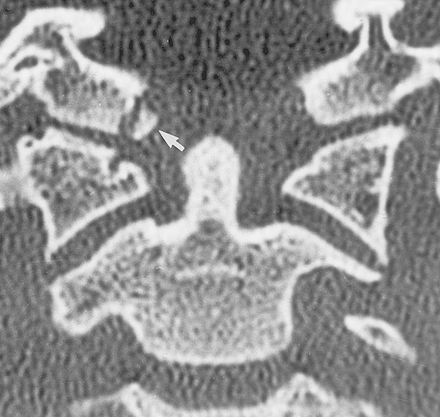


1. type II, transverse ligament
2. type I, alar ligament
3. type II, alar ligament
4. type III, alar ligament
5. type I, transverse ligament
6. **A 45 YO patient operated on for lumbar fusion comes back 3 weeks after with a wound leakage strongly suspected for infection. MR shows a deep site collection compatible with an abscess. How do you deal with it ? (NOT SUBMITTED TO CHAT GPT)**
7. Superficial samples, AB, wound follow-up
8. Surgical site revision with abscess’ drainage
9. Surgical site revision with abscess’ drainage and insertion of a suction tube
10. Surgical site revision with abscess’ drainage and insertion of a double-lumen suction tube with AB
11. Surgical site revision and local AB

**9. ASIA describes key muscles for upper limbs examination. Please add the corresponding nerve root**

Biceps brachialis: root…

Wrist extensor: root…

Triceps brachialis: root…

Finger flexors: root…

Little finger abductor: root…

ChatGPT

ASIA (American Spinal Injury Association) is a classification system used to assess and classify spinal cord injuries. It includes the evaluation of key muscles for the examination of upper limb function, along with their corresponding nerve roots. Here are some of the key muscles for upper limb examination according to ASIA, along with their associated nerve roots:

1. Elbow Flexors (Biceps Brachii, Brachialis): Nerve Root C5 and C6.
2. Elbow Extensors (Triceps Brachii): Nerve Root C7.
3. Wrist Extensors (Extensor Carpi Radialis Longus, Extensor Carpi Radialis Brevis): Nerve Root C6.
4. Wrist Flexors (Flexor Carpi Radialis): Nerve Root C7.
5. Finger Extensors (Extensor Digitorum, Extensor Digiti Minimi): Nerve Roots C7, C8.
6. Finger Flexors (Flexor Digitorum Profundus, Flexor Digitorum Superficialis): Nerve Roots C8, T1.
7. Intrinsic Hand Muscles (Interossei, Lumbricals): Nerve Roots T1, T2.

These muscles and their associated nerve roots are assessed during the examination of upper limb muscle strength and function in individuals with spinal cord injuries or other neurological conditions.

Please note that this is a simplified overview, and a comprehensive examination according to the ASIA classification involves a more detailed assessment of specific muscle groups and their innervation. A healthcare professional trained in ASIA evaluation should perform the examination for accurate assessment and interpretation of the findings.

1. **During the dissection phase of the abdominal musculature in a lateral thoracolumbar interbody fusion surgical approach, care must be taken not to cut which nerve in the superficial muscle layers before reaching the psoas:**

Ilioinguinal

Iliohypogastric

Subcostal nerve

Femoral nerve

ChatGPT

During the dissection phase of a lateral thoracolumbar interbody fusion (TLIF) surgical approach, care must be taken not to cut the iliohypogastric nerve in the superficial muscle layers before reaching the psoas muscle.

The iliohypogastric nerve is a sensory nerve that arises from the lumbar plexus, specifically from the ventral rami of spinal nerves L1. It courses through the abdominal wall and supplies sensory innervation to the skin of the lower abdomen, pubic region, and buttock.

In a lateral TLIF approach, the surgeon typically makes an incision in the lateral abdominal wall to access the spine. During the dissection, the superficial muscle layers are encountered before reaching the deeper structures such as the psoas muscle. Care must be taken to identify and preserve the iliohypogastric nerve, as cutting or damaging it can result in sensory deficits or neuropathic pain in the areas it innervates.

Preserving the iliohypogastric nerve is essential to minimize postoperative complications and ensure the preservation of normal sensation in the lower abdomen and associated regions. Surgeons performing lateral TLIF procedures should have a thorough understanding of the anatomy and take appropriate precautions to protect the iliohypogastric nerve during the dissection phase.

1. **Based on this image, what surgical approach (es) would be possible?**

**(NOT SUBMITTED TO CHAT GPT)**

1. ALIF
2. left XLIF
3. right XLIF
4. left OLIF
5. right OLIF


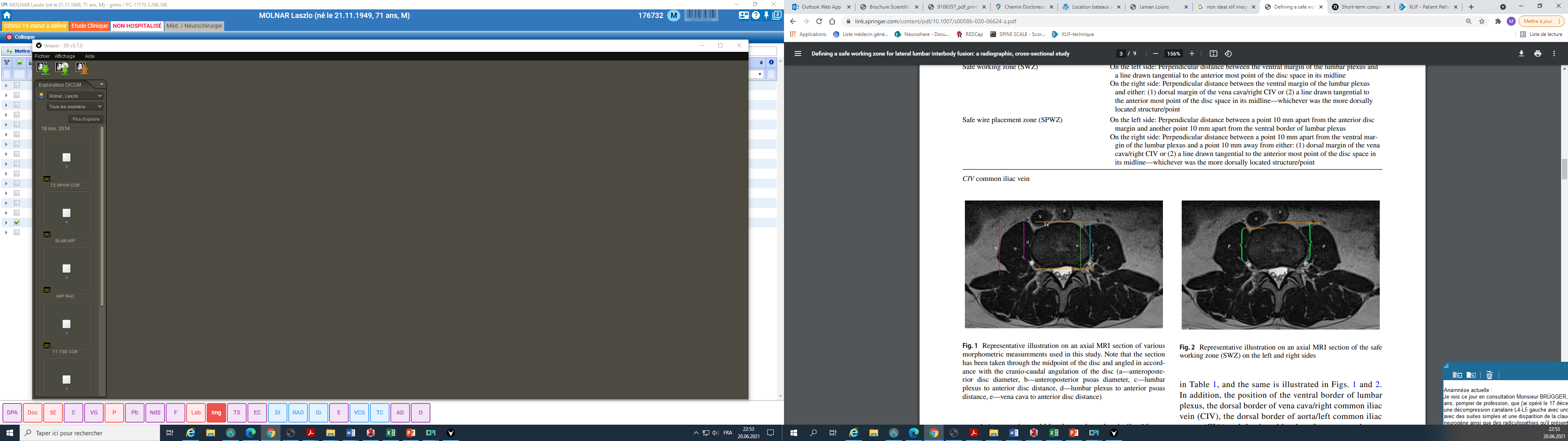


**12. Which of the following is correct for the TPA (T1 pelvic angle, or T1PA)**

is less accurate than the SVA (sagittal vertical axis) because it does not take into account the pelvic version

it’s ideal value should be less than 15°.

it’s ideal value should be greater than 20°

all these answers are wrong

ChatGPT

The correct answer is:

d) All these answers are wrong.

The provided options are not accurate descriptions of the T1 Pelvic Angle (TPA):

a) The TPA is a separate measurement from the Sagittal Vertical Axis (SVA) and evaluates different aspects of spinal alignment. While the SVA considers the vertical offset between the C7 plumb line and the posterior superior corner of the sacrum, the TPA focuses on the relationship between the T1 vertebral body and the pelvis.

b) and c) The ideal value of the TPA is not a fixed angle, but rather depends on various factors, including the individual's spinal and pelvic characteristics. There is no universally agreed-upon ideal value for the TPA. The TPA is interpreted in the context of the individual's overall spinal alignment and specific spinal condition being assessed.

It's important to consult with a qualified healthcare professional, such as a spine specialist or radiologist, for accurate interpretation of radiographic measurements like the TPA and to understand how they relate to an individual's specific case and condition.

**13. Which of the following is a major risk factor for moyamoya?**

Asian ancestry

Neurofibromatosis type II

Gorlin syndrome

Alpha-1 antitrypsin deficiency

smoking

ChatGPT

The correct answer is:

a) Asian ancestry

Moyamoya disease is a rare cerebrovascular disorder characterized by the progressive narrowing of certain blood vessels in the brain. While the exact cause of moyamoya disease is not fully understood, certain risk factors have been identified. One of the major risk factors for moyamoya disease is Asian ancestry. Moyamoya disease is more commonly seen in individuals of Asian descent, particularly in Japan and East Asian countries.

The other options listed (b) Neurofibromatosis type II, (c) Gorlin syndrome, (d) Alpha-1 antitrypsin deficiency, and (e) smoking, are not recognized as major risk factors for moyamoya disease. Neurofibromatosis type II and Gorlin syndrome are genetic conditions that may have neurological manifestations, but they are not directly associated with moyamoya disease. Similarly, Alpha-1 antitrypsin deficiency is a genetic disorder primarily affecting the lungs and liver. While smoking is a known risk factor for various cardiovascular and cerebrovascular diseases, it is not specifically associated with moyamoya disease.

1. **What is the pathology shown and its radiological sign (red circle) ?**

**(NOT SUBMITTED TO CHAT GPT)**


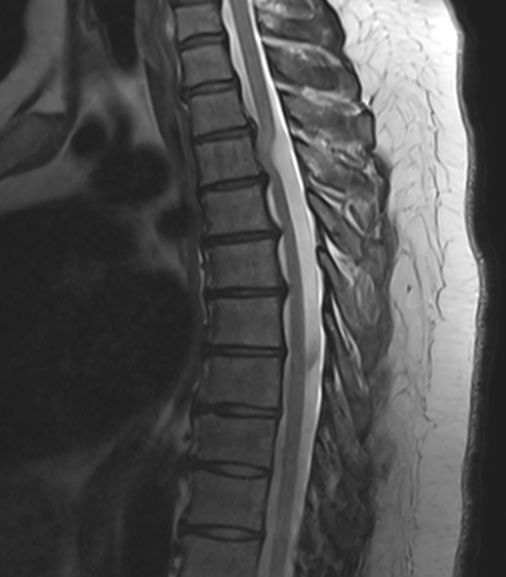


1. **This MRI belongs to a 65 year old male patient, with metastatic cancer of the prostate. He complains of mechanical pain, and he has no neurological deficit. There are mild signs of myelopathy from cord compression. The CT scan and MRI show a pathological fracture of T11.**

**How do you score this lesion according to the SINS- score system?**

**SINS is an abbreviation that stands for?**

**(NOT SUBMITTED TO CHAT GPT)**


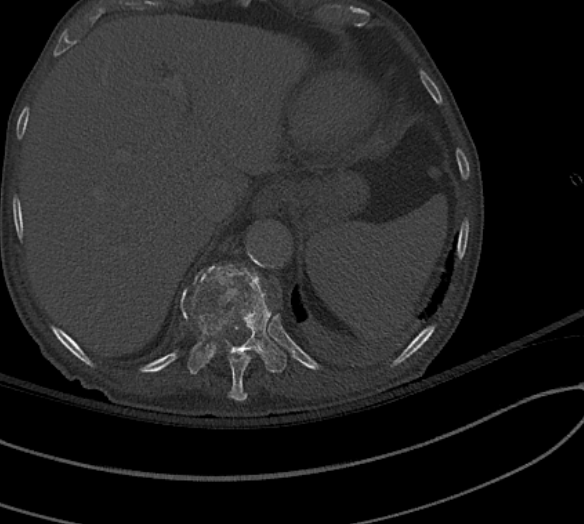

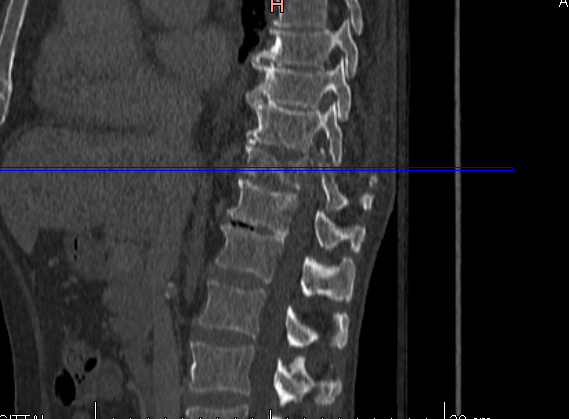


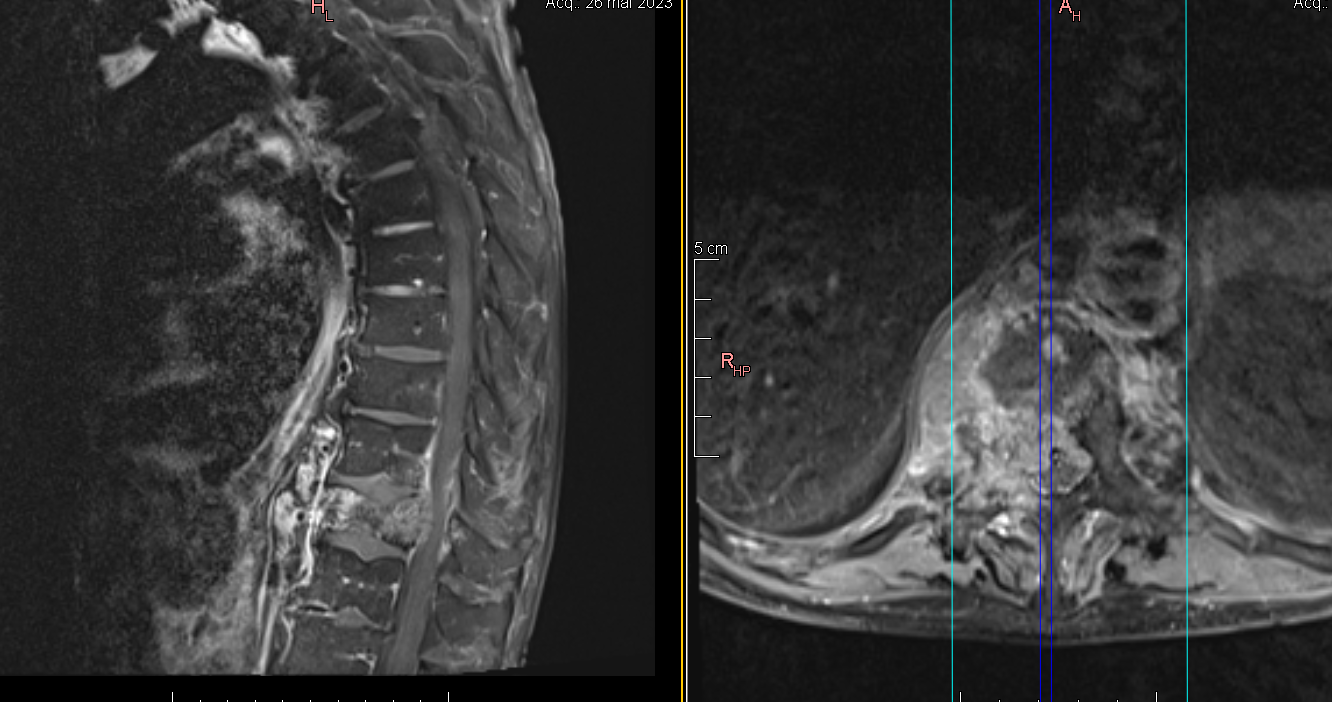


**16. Which statement about acute Epidural Hematoma (aEDH) is correct?**

They can cross the sutures of the skull

Mortality of aHED is normally lower than that of acute subdural hematoma (aHSD)

The risk of herniation is lower in younger patients if the hematoma is localised temporal

The cause of bleeding is typically a parenchymal laceration

ChatGPT

The correct statement about acute Epidural Hematoma (aEDH) is:

A) They can cross the sutures of the skull.

AEDH refers to the accumulation of blood between the dura mater (the outermost layer of the meninges) and the inner surface of the skull. The most common cause of aEDH is trauma, usually involving a skull fracture that damages the middle meningeal artery, leading to bleeding. Unlike other types of intracranial hemorrhage, such as acute subdural hematoma, aEDH has a unique characteristic of potentially crossing the sutures of the skull.

Option B is incorrect. The mortality of aEDH is generally higher than that of acute subdural hematoma (aHSD). AEDH often requires urgent surgical intervention to evacuate the hematoma and relieve intracranial pressure.

Option C is incorrect. The risk of herniation, a life-threatening complication where brain tissue is compressed and displaced, is higher in younger patients with localized temporal aEDH. The temporal lobe herniation can cause severe neurological deficits and is considered a medical emergency.

Option D is incorrect. The cause of bleeding in aEDH is typically arterial bleeding, often from the middle meningeal artery, rather than a parenchymal laceration.

Therefore, the correct statement is that acute Epidural Hematomas (aEDH) can cross the sutures of the skull (Option A).

**17. The most frequent site for a subependymoma is the…**

Third ventricle

Fourth ventricle

Left lateral ventricle

Right lateral ventricle

Septum pellucidum

ChatGPT

The correct answer is:

B) Fourth ventricle

Subependymomas are typically benign (non-cancerous) tumors that arise from the ependymal cells lining the ventricles of the brain or the central canal of the spinal cord. They are most commonly found in the fourth ventricle of the brain.

The fourth ventricle is located in the posterior fossa of the brain, between the brainstem and the cerebellum. It plays a role in the circulation of cerebrospinal fluid (CSF). Subependymomas can develop within the walls of the fourth ventricle, causing a mass effect and potentially leading to symptoms such as headache, hydrocephalus (excess fluid in the brain), or neurological deficits.

While subependymomas can rarely occur in other locations, such as the lateral ventricles or the septum pellucidum (Option E), the fourth ventricle (Option B) is the most frequent site for subependymomas.

**18. Pineal calcifications are considered abnormal if encountered in patients younger than**

6 years

12 years

18 years

26 years

30 years

ChatGPT

Pineal calcifications are considered abnormal if encountered in patients younger than:

C) 18 years

Pineal calcifications are commonly encountered on imaging studies such as CT (computed tomography) or MRI (magnetic resonance imaging). They are small, calcified structures within the pineal gland, a small gland located in the brain that plays a role in the regulation of sleep-wake cycles and hormone production.

Pineal calcifications are typically found in individuals as they age and are considered a normal finding in adults. However, the presence of pineal calcifications in patients younger than 18 years is considered abnormal and may warrant further investigation.

Therefore, if pineal calcifications are encountered in patients younger than 18 years (Option C), it is important to evaluate the individual further to determine the underlying cause and rule out any potential abnormalities or pathologies.

**19. Which segment is delimited by the callosomarginal sulcus and the origin of the precuneal artery?**

A3

M3

A4

C1

M4

ChatGPT

The segment delimited by the callosomarginal sulcus and the origin of the precuneal artery is:

C) A4

In neuroanatomy, the segments of cerebral arteries are often classified based on their relationship to specific sulci or landmarks. The callosomarginal sulcus, also known as the cingulate sulcus or the sulcus marginalis, is a prominent sulcus located on the medial surface of the cerebral hemisphere. It separates the frontal lobe from the cingulate gyrus.

The precuneal artery is a branch of the posterior cerebral artery (PCA) that supplies blood to the precuneus, a region of the parietal lobe located on the medial surface of the cerebral hemisphere.

The segment between the callosomarginal sulcus and the origin of the precuneal artery is referred to as A4. This segment represents a portion of the anterior cerebral artery (ACA), which is responsible for supplying blood to the medial surfaces of the cerebral hemispheres.

Therefore, the correct answer is C) A4.

20. The carotid cistern (or optic-carotid cistern) is a key landmark for aneurysm surgery as it allows CSF drainage to relax brain parenchyma and a proximal control over the supraclinoid segment of the internal carotid artery. Which statement is correct about the anatomy of the carotid cistern? A) Optic nerve lies medially and below the intradural carotid artery. B) Intradural carotid artery is lateral to the ipsilateral optic nerve and the sylvian fissure lies just laterally to the carotid cistern. C) Liliquist membrane corresponds to folds of arachnoid layers between the carotid artery and the optic nerve and lies laterally. D) The optic nerve is in close contact with the medial aspect of the choroidal segment of the carotid artery. E) Posterior clinoid covers the supero-lateral aspect of the internal carotid artery and corresponds to the roof of the carotid cistern.

ChatGPT

The correct statement about the anatomy of the carotid cistern is:

B) Intradural carotid artery is lateral to the ipsilateral optic nerve, and the sylvian fissure lies just laterally to the carotid cistern.

The carotid cistern, also known as the optic-carotid cistern, is an anatomical space located in the subarachnoid space surrounding the internal carotid artery (ICA) and the optic nerve. It is an important landmark for aneurysm surgery due to its proximity to the supraclinoid segment of the ICA.

In the carotid cistern anatomy, the intradural carotid artery is positioned laterally to the ipsilateral optic nerve. The optic nerve lies medially and below the intradural carotid artery, as stated in option A, is incorrect. The correct relationship is that the carotid artery is lateral to the optic nerve.

The sylvian fissure, a prominent fissure that separates the frontal and temporal lobes, lies just laterally to the carotid cistern. This relationship is correctly described in option B.

Option C is incorrect because Liliequist membrane refers to a different anatomical structure. It is a fold of arachnoid membrane that separates the interpeduncular cistern from the ambient cistern and has no direct association with the carotid cistern.

Option D is incorrect because the optic nerve does not have direct contact with the medial aspect of the choroidal segment of the carotid artery.

Option E is incorrect because the posterior clinoid process does not cover the supero-lateral aspect of the internal carotid artery. It is a bony projection located posteriorly to the carotid cistern, but it does not form the roof of the carotid cistern.

Therefore, the correct statement is that the intradural carotid artery is lateral to the ipsilateral optic nerve, and the sylvian fissure lies just laterally to the carotid cistern (Option B).

**21. In glioblastomas which molecular alteration is associated with a better prognosis (as in better response to treatment)**

IDH-1 wild-type

ATRX mutation

p53 mutation

MGMT methylation

1p19q co-deletion

ChatGPT

In glioblastomas, the molecular alteration associated with a better prognosis and better response to treatment is:

D) MGMT methylation

Glioblastoma is an aggressive type of brain tumor with a poor prognosis. However, certain molecular alterations can provide prognostic information and predict the response to treatment. MGMT (O-6-methylguanine-DNA methyltransferase) is a DNA repair enzyme that can influence the tumor's response to chemotherapy, particularly to alkylating agents such as temozolomide.

MGMT methylation refers to the addition of a methyl group to the MGMT gene promoter region, which leads to the silencing of the gene. Glioblastoma patients with MGMT promoter methylation tend to have a better response to treatment with temozolomide and generally have improved overall survival compared to patients without MGMT methylation.

The other molecular alterations listed are not associated with a better prognosis or treatment response in glioblastomas:

A) IDH-1 wild-type: Glioblastomas often have wild-type IDH-1, but IDH-1 mutations are more commonly found in lower-grade gliomas and are associated with a better prognosis in those cases. In glioblastomas, wild-type IDH-1 is the typical molecular profile.

B) ATRX mutation: ATRX mutations are more commonly associated with lower-grade gliomas, such as astrocytomas or oligodendrogliomas. In glioblastomas, ATRX mutations are less frequent and not associated with a better prognosis.

C) p53 mutation: p53 mutations are common in many cancers, including glioblastomas. However, p53 mutations in glioblastomas are not associated with a better prognosis. They are often linked to the aggressive nature of the tumor.

E) 1p19q co-deletion: 1p19q co-deletion is a molecular alteration found in oligodendrogliomas, a distinct subtype of glioma. It is associated with a better prognosis in oligodendrogliomas, but it is not typically seen in glioblastomas.

Therefore, the correct answer is D) MGMT methylation.

**22. Which artery is anatomically closely related to the VII and VIII cranial nerve?**

Posterior inferior cerebellar artery

Superior cerebellar artery

Anterior inferior cerebellar artery

Vertebral artery

Basilar artery

ChatGPT

The artery that is anatomically closely related to the VII (facial) and VIII (vestibulocochlear) cranial nerves is:

C) Anterior inferior cerebellar artery

The VII and VIII cranial nerves are located in the posterior cranial fossa of the brain. The anterior inferior cerebellar artery (AICA) is a branch of the basilar artery, and it runs in close proximity to the VII and VIII cranial nerves as it supplies blood to the structures in that region.

The AICA wraps around the brainstem, and its branches supply the anterior inferior portion of the cerebellum, including the flocculonodular lobe, the lateral pons, and the cochlear and vestibular nuclei. The proximity of the AICA to the VII and VIII cranial nerves makes it an important landmark during surgical procedures or when assessing potential sources of vascular compression or pathology in this area.

Option A, the posterior inferior cerebellar artery (PICA), supplies the posterior inferior part of the cerebellum and does not directly relate to the VII and VIII cranial nerves.

Option B, the superior cerebellar artery (SCA), is another branch of the basilar artery, but it runs above the VII and VIII cranial nerves and primarily supplies the superior surface of the cerebellum.

Option D, the vertebral artery, is one of the two main arteries that join to form the basilar artery. While it contributes to the blood supply of the posterior fossa, it does not have a direct anatomical relationship with the VII and VIII cranial nerves.

Option E, the basilar artery, is formed by the union of the two vertebral arteries and runs along the anterior surface of the brainstem. While it provides blood supply to various structures in the posterior fossa, it is not as closely related to the VII and VIII cranial nerves as the AICA.

Therefore, the correct answer is C) Anterior inferior cerebellar artery (AICA).

**23. The caudate nucleus forms:**

The medial wall of the frontal horn

The floor of the lateral horn

The lateral wall of the occipital horn

The roof of the lateral ventricle

None of the above

ChatGPT

The caudate nucleus does not form any of the structures mentioned in the options. Therefore, the correct answer is:

E) None of the above.

The caudate nucleus is a C-shaped structure located deep within the cerebral hemispheres, specifically within the basal ganglia. It is involved in various functions, including motor control, reward processing, and cognitive functions.

While the caudate nucleus is close to the lateral ventricles, it does not form any of the specific structures mentioned in the options. It is important to note that the lateral ventricles are fluid-filled spaces within the brain, and their structures are mainly formed by the surrounding walls, such as the corpus callosum, fornix, and thalamus.

Therefore, the caudate nucleus does not form the medial wall of the frontal horn (Option A), the floor of the lateral horn (Option B), the lateral wall of the occipital horn (Option C), or the roof of the lateral ventricle (Option D).

**24. What happened here?**

- **Name the procedure.**
- **Explain the differences between the main two surgical techniques.**
- **Explain displayed modifications (A B C D E )**

**(NOT SUBMITTED TO CHAT GPT)**


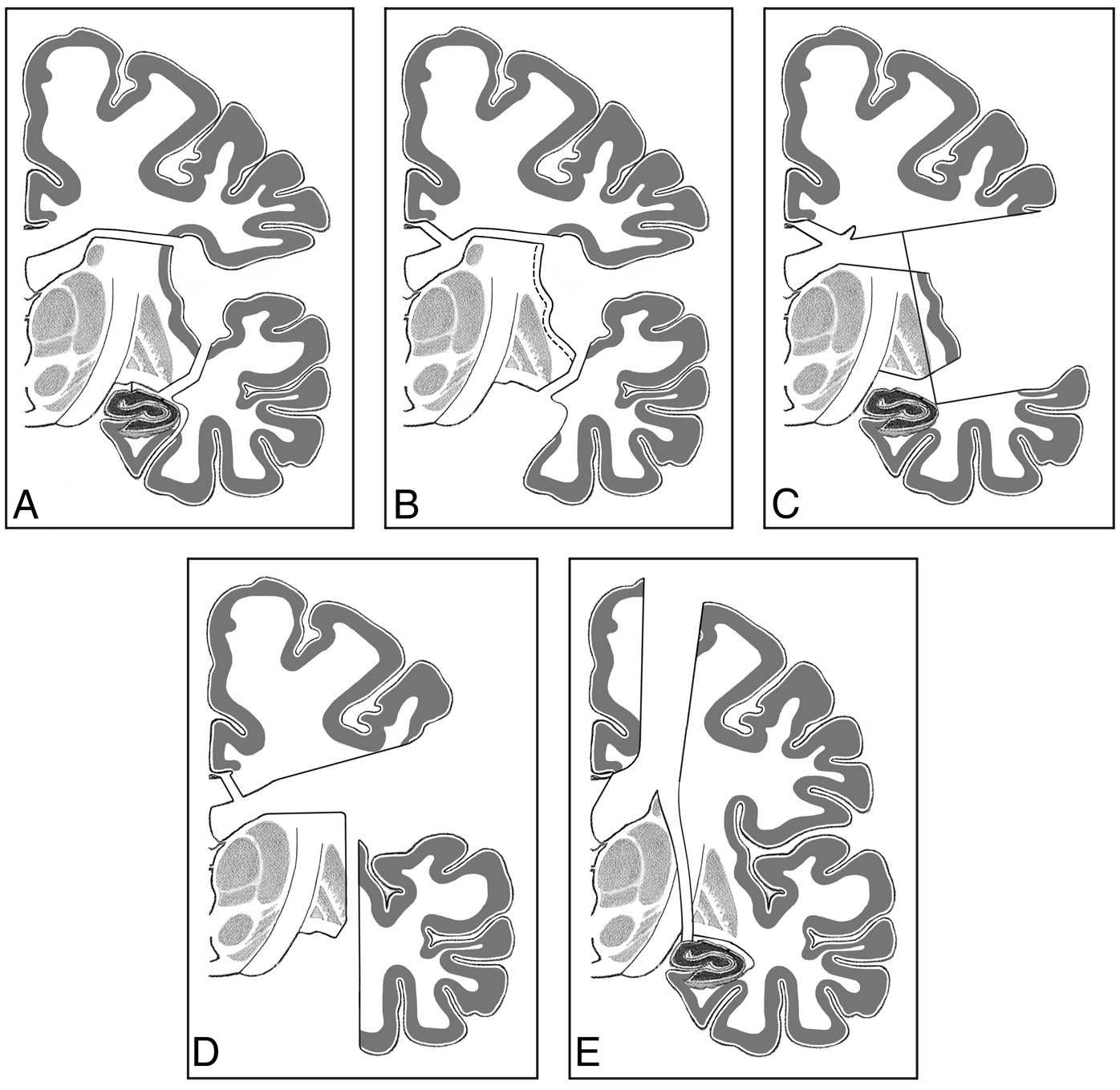


**25. Explain the concept of multisensory integration for the internal organization of human self-consciousness – and name at least one anatomical region substrate**

ChatGPT

Multisensory integration refers to the process by which the brain combines information from different sensory modalities, such as vision, hearing, touch, and proprioception, to create a unified perception of the world. It plays a crucial role in the internal organization of human self-consciousness by integrating sensory inputs and creating a coherent sense of body ownership, self-location, and self-identity.

An important anatomical region substrate involved in multisensory integration and self-consciousness is the temporo-parietal junction (TPJ). The TPJ is located at the intersection of the temporal and parietal lobes, and it is involved in processing sensory information related to body perception, spatial awareness, and social cognition.

Studies have shown that the TPJ plays a key role in integrating visual, auditory, and somatosensory inputs to create a unified sense of self. It receives information from multiple sensory modalities and combines them to generate a coherent representation of the body and its relation to the external world. For example, when we see an object and simultaneously feel its touch, the TPJ integrates visual and tactile information to create a unified perception of the object's properties and our own interaction with it.

Furthermore, disruptions or abnormalities in the TPJ have been associated with alterations in self-consciousness, such as out-of-body experiences and feelings of disembodiment. Dysfunction in multisensory integration processes within the TPJ can lead to disturbances in the sense of self and body ownership.

Other anatomical regions involved in multisensory integration and self-consciousness include the superior temporal gyrus (STG), insular cortex, and prefrontal cortex. These regions are interconnected and work together to process and integrate sensory inputs, contributing to our sense of self and awareness of the external world.

Overall, multisensory integration, facilitated by regions like the TPJ, is a fundamental process for the internal organization of human self-consciousness. It allows us to create a coherent and unified sense of self by integrating information from various sensory modalities.

**26. What may be the cause/s for rebleeding following resection of a cerebral AVM. You may include a pathophysiological theory**

ChatGPT

Rebleeding following resection of a cerebral arteriovenous malformation (AVM) can occur due to various factors and underlying pathophysiological mechanisms. Some potential causes for rebleeding include:

1. Residual AVM: Incomplete resection of the AVM may leave behind residual abnormal blood vessels that can continue to bleed. This can occur due to the complex and intricate nature of AVMs, with intricate connections between arteries and veins that may be challenging to completely remove during surgery.
2. Fragile vessels and vascular remodeling: AVMs are characterized by abnormal and fragile blood vessels. Surgical resection can disrupt the abnormal vascular network, leading to vessel wall injury and subsequent bleeding. Additionally, the process of vascular remodeling can occur following AVM resection, where the vessels undergo changes in response to the altered hemodynamic conditions. These remodeling processes may be associated with vessel fragility and subsequent rebleeding.
3. Hemodynamic changes: Resection of an AVM alters the local hemodynamic environment by eliminating the abnormal high-flow shunting of blood. This sudden change in blood flow dynamics can result in hemodynamic stress on the remaining vessels, potentially leading to vessel rupture and rebleeding.
4. Venous hypertension: AVMs often cause venous hypertension due to the abnormal direct connection between arteries and veins. Resection of the AVM can lead to alterations in the venous drainage pattern, potentially resulting in increased venous pressure. Elevated venous pressure can strain the fragile vessels and increase the risk of rebleeding.
5. Delayed angiogenesis: Following AVM resection, there is a process of angiogenesis and vessel remodeling in the surrounding brain tissue. This angiogenic response can result in the development of new vessels, which may be fragile and prone to bleeding.

The pathophysiological theory behind rebleeding following AVM resection revolves around the disruption of the abnormal vascular network, vessel wall injury, and subsequent changes in hemodynamics and vessel remodeling. The fragile nature of the vessels, combined with altered blood flow dynamics, can increase the risk of rebleeding even after surgical intervention.

It is important to note that the risk of rebleeding following AVM resection is multifactorial and varies depending on the specific characteristics of the AVM, the surgical technique employed, and individual patient factors. Close postoperative monitoring and appropriate management strategies are crucial to minimize the risk of rebleeding and optimize patient outcomes.

**27. What are arguments in favor of surgery (with or without presurgical embolization) for a cerebral AVM**

ChatGPT

There are several arguments in favor of surgical intervention, with or without presurgical embolization, for a cerebral arteriovenous malformation (AVM). These arguments consider the potential benefits of surgery in managing AVMs and the specific circumstances of each case. Some of the arguments in favor of surgery for a cerebral AVM include:

1. Risk of hemorrhage: AVMs have the potential to rupture and cause intracranial hemorrhage, which can lead to severe neurological deficits or even death. Surgical resection aims to eliminate the AVM and reduce the risk of future hemorrhage. By removing the abnormal vessels and restoring normal blood flow, surgery can provide long-term protection against bleeding.
2. Symptom relief: AVMs can cause symptoms such as seizures, headaches, neurological deficits, or cognitive impairments. Surgery can alleviate these symptoms by removing the AVM and resolving the underlying vascular abnormality. This can lead to improved quality of life and functional outcomes for patients.
3. Prevention of complications: AVMs can lead to other complications such as venous congestion, venous hypertension, or vascular steal phenomena. Surgical removal of the AVM can help prevent these complications by restoring normal blood flow patterns and reducing the burden on the surrounding brain tissue.
4. Curative potential: Surgical resection offers the potential for a curative treatment for AVMs. Complete removal of the AVM eliminates the risk of future hemorrhage and related complications. In some cases, surgical intervention may provide the best chance of achieving a complete cure and long-term freedom from AVM-related issues.
5. Preservation of normal brain tissue: Advances in neurosurgical techniques and imaging technologies have improved the ability to precisely locate and remove AVMs while minimizing damage to surrounding healthy brain tissue. Preservation of normal brain tissue is an important consideration in surgical planning and can contribute to better postoperative outcomes.
6. Adjunctive therapies: Surgery can be combined with other treatment modalities, such as presurgical embolization or postoperative radiosurgery, to enhance the overall effectiveness of AVM management. Presurgical embolization can reduce the size or vascularity of the AVM, making it more amenable to surgical resection. These multimodal approaches can be tailored to the specific characteristics of the AVM and individual patient factors, optimizing treatment outcomes.

It is important to note that the decision to pursue surgery for a cerebral AVM should be made after a comprehensive evaluation of the patient's specific case, considering factors such as AVM location, size, angioarchitecture, and the patient's overall health. Multidisciplinary discussions involving neurosurgeons, interventional neuroradiologists, and neurologists are crucial in determining the most appropriate treatment strategy for each individual patient.

**28. Which statements are true about the cerebral perfusion pressure (CPP)?**

is constant

equals the difference between mean arterial pressure and intracranial pressure

correlates with the cerebral perfusion

cerebral vascular resistance (vasoreactivity) autoregulates the cerebral perfusion

ChatGPT

Of the given statements about cerebral perfusion pressure (CPP), the following statements are true:

B) CPP equals the difference between mean arterial pressure (MAP) and intracranial pressure (ICP). CPP is calculated by subtracting ICP from MAP. It represents the pressure gradient driving blood flow to the brain and is an important parameter for assessing cerebral perfusion.

D) Cerebral vascular resistance (vasoreactivity) autoregulates the cerebral perfusion. The brain has the ability to regulate its own blood flow through a mechanism called autoregulation. This mechanism ensures that blood flow to the brain remains relatively constant over a range of systemic blood pressures. Cerebral vascular resistance adjusts to changes in blood pressure to maintain a stable CPP and adequate cerebral perfusion.

The other statements are not true:

A) CPP is not constant. It can vary depending on changes in MAP and ICP. Fluctuations in MAP and ICP can lead to changes in CPP, which can impact cerebral perfusion.

C) CPP does not directly correlate with cerebral perfusion. While CPP is an important determinant of cerebral blood flow, it is not the sole factor. Other factors, such as cerebrovascular resistance, regional vascular autoregulation, and metabolic demands, also play a role in regulating cerebral perfusion.

In summary, the true statements regarding CPP are that it equals the difference between MAP and ICP (Statement B) and that cerebral vascular resistance autoregulates cerebral perfusion (Statement D).

**29. Intracranial pressure (one correct answer):**

Is constant

Varies more according to head position

Varies more according to respiration

Varies more according to the arterial pressure

ChatGPT

Intracranial pressure (ICP) varies more according to the arterial pressure.

Option D) "Varies more according to the arterial pressure" is the correct answer. Intracranial pressure is influenced by various factors, including arterial pressure, cerebrovascular resistance, and venous pressure. Changes in arterial pressure can directly affect ICP, with increases in arterial pressure leading to increased ICP and vice versa. Fluctuations in arterial pressure, such as during systemic hypertension or hypotension, can result in corresponding changes in intracranial pressure.

**30. Name the 4 most common fetal variants of intracranial arteries:**

ChatGPT

The four most common fetal variants of intracranial arteries are:

1. Fetal posterior cerebral artery (fPCA) variant: In this variant, the posterior cerebral artery arises from the internal carotid artery instead of the basilar artery. It is also known as persistent fetal origin of the posterior cerebral artery (pFOPA).
2. Fetal origin of the posterior communicating artery (fPCA): In this variant, the posterior communicating artery arises from the internal carotid artery instead of the posterior cerebral artery.
3. Fetal middle cerebral artery (fMCA) variant: In this variant, the middle cerebral artery has a different pattern of branching or arises from a different location than the typical adult configuration. This variant is also referred to as persistent trigeminal artery (PTA).
4. Fetal origin of the anterior cerebral artery (fACA): In this variant, the anterior cerebral artery arises from the internal carotid artery instead of the anterior communicating artery or the typical bifurcation of the internal carotid artery.

These fetal variants of intracranial arteries can be detected on imaging studies such as cerebral angiography or magnetic resonance angiography (MRA). It is important to identify these variants as they can have implications for surgical planning and treatment of cerebrovascular diseases.

**31. Grading of the cerebral arteriovenous malformation is useful to:**

Assess the risk of bleeding with the Spetzler-Martin grading system TRUE / FALSE

Assess more easily the surgical risk (both mortality and morbidity) TRUE / FALSE

with the Spetzler-Martin grading system

Assess more precisely the surgical risk (both mortality and morbidity) TRUE / FALSE

with the Catapano grading system

Predict the outcome of patients treated by radiosurgery TRUE / FALSE

with the Pollock grading system

ChatGPT

The statements can be evaluated as follows:

A) Assess the risk of bleeding with the Spetzler-Martin grading system: TRUE. The Spetzler-Martin grading system is widely used to assess the risk of bleeding associated with cerebral arteriovenous malformations (AVMs). This grading system takes into account factors such as AVM size, location, and venous drainage pattern to provide an estimation of the risk of hemorrhage. Higher grades on the Spetzler-Martin scale are associated with a higher risk of bleeding.

B) Assess more easily the surgical risk (both mortality and morbidity) with the Spetzler-Martin grading system: TRUE. The Spetzler-Martin grading system is primarily designed to assess the surgical risk associated with AVM resection. It takes into consideration AVM characteristics such as size, eloquence of the surrounding brain, and venous drainage to estimate the surgical morbidity and mortality risks. Higher grades on the Spetzler-Martin scale indicate a higher surgical risk.

C) Assess more precisely the surgical risk (both mortality and morbidity) with the Catapano grading system: FALSE. The Catapano grading system is not commonly used for assessing the surgical risk of AVM resection. The Spetzler-Martin grading system is the more widely recognized and utilized grading system for surgical risk assessment in AVM cases.

D) Predict the outcome of patients treated by radiosurgery with the Pollock grading system: TRUE. The Pollock grading system is specifically designed to predict the outcome of patients treated with radiosurgery for AVMs. It considers factors such as AVM size, location, and patient age to estimate the likelihood of complete obliteration of the AVM after radiosurgery and the risk of post-treatment hemorrhage. Higher grades on the Pollock scale are associated with a lower chance of complete AVM obliteration and a higher risk of post-treatment hemorrhage.

In summary, the correct statements are:

A) Assess the risk of bleeding with the Spetzler-Martin grading system: TRUE. B) Assess more easily the surgical risk (both mortality and morbidity) with the Spetzler-Martin grading system: TRUE. D) Predict the outcome of patients treated by radiosurgery with the Pollock grading system: TRUE.

**32. Intermediate ventral nucleus (VIM) of the thalamus is a relay for efferent fibers coming from**

Cerebellum

Internal pallidum

ChatGPT

The intermediate ventral nucleus (VIM) of the thalamus serves as a relay for efferent fibers coming from the cerebellum. Therefore, option A) "Cerebellum" is the correct answer. The VIM is involved in the motor control circuitry and plays a crucial role in the treatment of movement disorders such as essential tremor and Parkinson's disease through deep brain stimulation.

**33**. **Look carefully at this picture (NOT SUBMITTED TO CHAT GPT)**


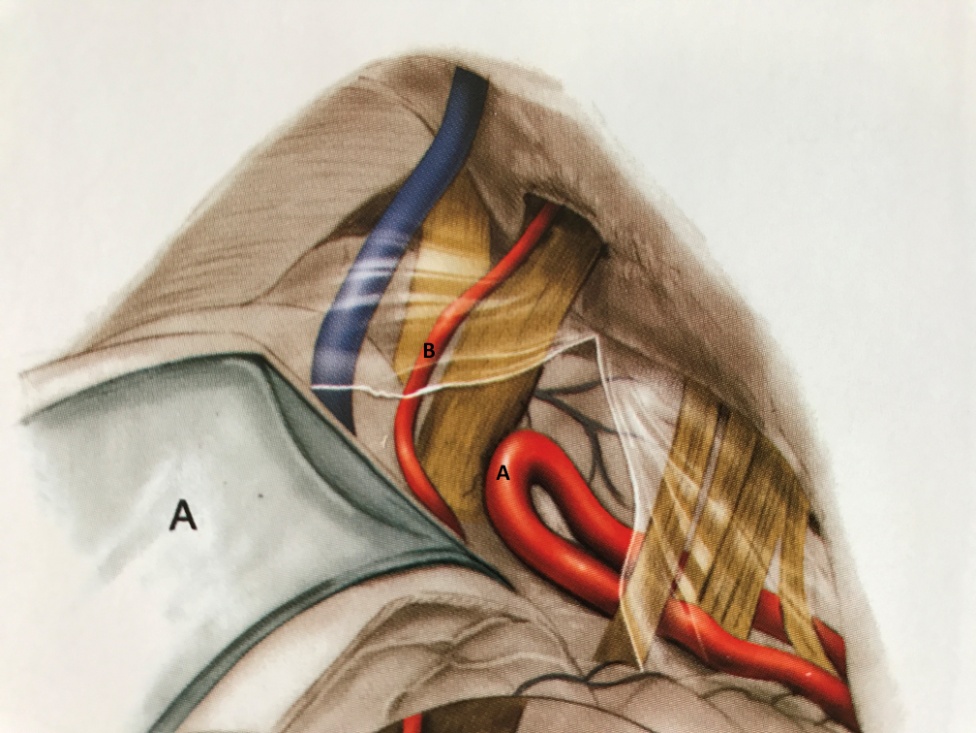


1. What is (most likely) the artery conflicting with this right facial nerve ?
2. What is the name of the artery labeled by “B” ?
3. Artery “B” is often a branch of another artery. Which one?

**34**. **Look carefully at this picture**


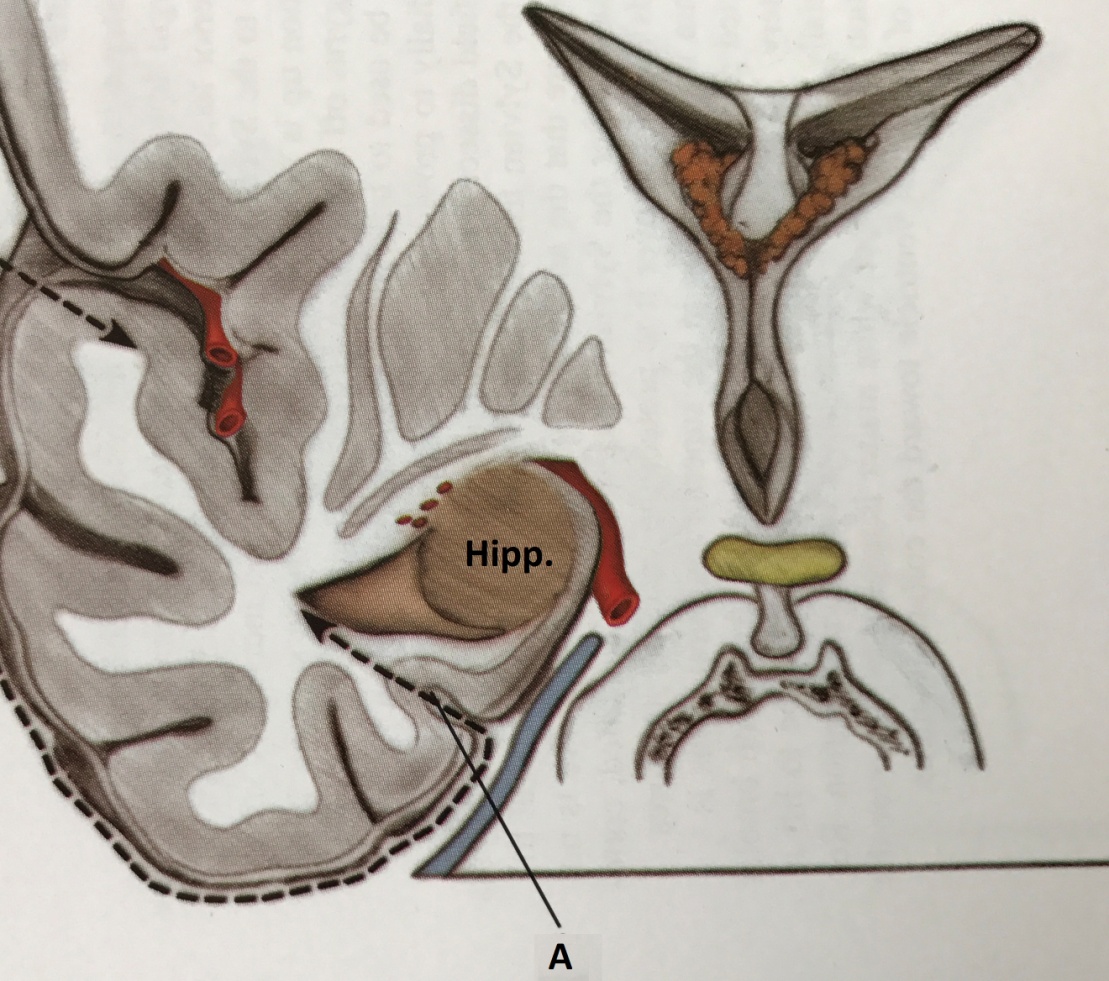


**Right temporal lobe, coronal view. Hipp: hippocampus. Name the sulcus lableded “A”, that is an important landmark during anterior temporal lobectomy. (NOT SUBMITTED TO CHAT GPT)**

**35. What is the usually recommended posterior extension of an anterior temporal lobectomy for epilepsy surgery on the right side? (Starting from the temporal pole)**

3.5 cm

4.5 cm

5.5 cm

ChatGPT

The usually recommended posterior extension of an anterior temporal lobectomy for epilepsy surgery on the right side, starting from the temporal pole, is approximately 4.5 cm. Therefore, option B) "4.5 cm" is the correct answer. This extension allows for the removal of the mesial structures of the temporal lobe, including the hippocampus and amygdala, which are commonly involved in temporal lobe epilepsy. The specific extent of resection may vary depending on individual patient characteristics and the surgeon's judgment. It is important to note that surgical decisions and techniques should be made by a qualified neurosurgeon based on the patient's specific condition and preoperative evaluation.

**36. Name in the right order the steps of meningioma surgery:**

Dissect – Debulk – Devascularize

Devascularize – Debuk – Dissect

Debulk – Dissect – Devascularize

Debulk – Devascularize – Dissect

ChatGPT

The correct order of steps for meningioma surgery is:

C) Debulk – Dissect – Devascularize

The typical sequence of surgical steps for meningioma removal involves debulking the tumor, followed by careful dissection to separate it from surrounding structures, and then devascularizing or controlling the blood supply to the tumor. This order helps to minimize bleeding and facilitate safer tumor removal.

**37. Name the most common bacteria involved in spondylodiscitis:**

ChatGPT

The most common bacteria involved in spondylodiscitis include:

1. Staphylococcus aureus: This bacterium is the most common cause of spondylodiscitis, particularly in adults. It can be either methicillin-sensitive Staphylococcus aureus (MSSA) or methicillin-resistant Staphylococcus aureus (MRSA).
2. Streptococcus species: Streptococcus species, including Streptococcus pneumoniae and Streptococcus pyogenes, are also commonly associated with spondylodiscitis, especially in children and elderly individuals.
3. Escherichia coli: This Gram-negative bacterium, commonly found in the gastrointestinal tract, can cause spondylodiscitis, particularly in older individuals or those with urinary tract infections.
4. Pseudomonas aeruginosa: Pseudomonas aeruginosa is a less common but significant cause of spondylodiscitis, typically associated with immunocompromised individuals or those with intravenous drug use.

Other less common pathogens that can cause spondylodiscitis include other Gram-positive cocci (such as coagulase-negative staphylococci), anaerobic bacteria, Mycobacterium tuberculosis (in tuberculosis-associated cases), and fungal infections (such as Candida or Aspergillus).

It's important to note that the specific causative bacteria may vary depending on factors such as patient age, underlying comorbidities, immune status, and geographic location. Diagnosis and treatment should be guided by the individual patient's clinical presentation, imaging findings, and appropriate microbiological cultures.

**38. What is the most common intramedullary cervical tumor?**

Meningioma

Ependymoma

Metastasis

Glioblastoma

Schwannoma

ChatGPT

The most common intramedullary cervical tumor is B) Ependymoma. Ependymomas are glial cell-derived tumors that commonly arise within the spinal cord, particularly in the cervical region. They account for the majority of intramedullary tumors in the cervical spine. Ependymomas can occur at any age but are most commonly seen in children and young adults. They typically present with symptoms related to spinal cord compression and can cause neurological deficits. Proper diagnosis and treatment of intramedullary cervical tumors require imaging studies, such as MRI, and may involve surgical resection or other treatment modalities based on the individual patient's condition.

**39. A 27 yo male presents with back pain, no neurological deficit. Pain is more intense over night and responds to acetylsalicylic acid. There is no history of trauma nor infection. T-spine CT scan shows a bony lesion within the R T10 pedicle. You suspect a tumor. Which one is most likely to occur?**

Chordoma

Metastasis

Osteoid osteoma

Ewing Sarcoma

Osteoblastoma

ChatGPT

In the given clinical scenario, the most likely tumor to occur in the right T10 pedicle of a 27-year-old male with back pain that intensifies at night and responds to acetylsalicylic acid is E) Osteoid osteoma.

Osteoid osteoma is a benign bone tumor that commonly presents with localized bone pain that is often worse at night and relieved by nonsteroidal anti-inflammatory drugs (such as acetylsalicylic acid). It typically affects younger individuals and can be found in various bones, including the spine. The characteristic radiographic finding of osteoid osteoma is a central nidus surrounded by reactive sclerotic bone.

While the other tumor options listed (chordoma, metastasis, Ewing sarcoma, and osteoblastoma) can occur in the spine, the clinical presentation and response to acetylsalicylic acid in this case are more suggestive of osteoid osteoma. However, further diagnostic evaluation, such as biopsy or additional imaging studies, would be necessary to confirm the diagnosis.

**40. Which of the following nerves is part of the lumbar plexus?**

Iliohypogastric n.

Pudendal n.

Obturator n.

External popliteal n.

Ilioinguinal n.

ChatGPT

Among the nerves listed, the one that is part of the lumbar plexus is C) Obturator nerve.

The lumbar plexus is a network of nerves formed by the anterior rami of the lumbar spinal nerves (L1-L4). It supplies motor and sensory innervation to various structures of the lower limb. The obturator nerve arises from the anterior divisions of the L2-L4 spinal nerves and runs through the psoas muscle before emerging near the pelvic region. It innervates the muscles of the medial compartment of the thigh and provides sensory innervation to the skin of the medial thigh and knee.

The other nerves listed are not part of the lumbar plexus: A) Iliohypogastric nerve: Arises from the L1 spinal nerve and is part of the thoracoabdominal nerves. B) Pudendal nerve: Arises from the sacral plexus (S2-S4) and innervates the perineum and external genitalia. D) External popliteal nerve (common fibular nerve): Arises from the sacral plexus (L4-S2) and supplies motor and sensory innervation to the anterior and lateral compartments of the leg and the dorsum of the foot. E) Ilioinguinal nerve: Arises from the first lumbar nerve (L1) and is part of the thoracoabdominal nerves.

**41. Connect each technique to the appropriate statement**

a) OLIF 1) has a risk of retrograde ejaculation

b) ALIF 2) allows exposure of the Kambin triangle

c) XLIF 3) is an “anterior to psoas” technique (ATP)

d) TLIF 4) is a “transpsoas” technique

ChatGPT

The correct matching of the surgical approaches and their respective descriptions is as follows:

a) OLIF 3) is an "anterior to psoas" technique (ATP) b) ALIF 2) allows exposure of the Kambin triangle c) XLIF 4) is a "transpsoas" technique d) TLIF 1) has a risk of retrograde ejaculation

Explanation:

- OLIF (Oblique Lateral Interbody Fusion) is an anterior to psoas technique (ATP) that involves accessing the spine through an oblique lateral approach, passing through the retroperitoneal space.
- ALIF (Anterior Lumbar Interbody Fusion) allows exposure of the Kambin triangle, which is a region between the exiting nerve root and traversing nerve root in the intervertebral foramen.
- XLIF (eXtreme Lateral Interbody Fusion) is a transpsoas technique that accesses the spine through a lateral approach, passing through the psoas muscle.
- TLIF (Transforaminal Lumbar Interbody Fusion) has a risk of retrograde ejaculation. This technique involves accessing the spine through a posterior approach, entering the intervertebral disc space through the neural foramen.

It's important to note that each surgical approach has its own specific indications, advantages, and potential risks, and the choice of approach depends on various factors such as the patient's condition, the level of spinal pathology, and surgeon preference.

**42. About hyponatremia, which of the following statements is true?**

Water restriction always allows to treat all hyponatremia

It is always related to a syndrome of inappropriate secretion of antidiuretic hormone (SIADH)

If it is related to a cerebral wasting syndrome, water restriction is the key treatment to treat the hyponatremia

Hyponatremia during SIADH is usually hyposmolar and patients have elevated urinary osmolarity

Urinary output measurement and blood test are recommended but are not essential

ChatGPT

Among the given options, the true statement about hyponatremia is:

D) Hyponatremia during SIADH is usually hyposmolar, and patients have elevated urinary osmolarity.

Explanation:

- A) Water restriction alone may not always be sufficient to treat all cases of hyponatremia. The treatment approach depends on the underlying cause and severity of the condition.
- B) Hyponatremia can be caused by various factors, and it is not always related to the syndrome of inappropriate secretion of antidiuretic hormone (SIADH). Other conditions such as fluid overload, adrenal insufficiency, or certain medications can also cause hyponatremia.
- C) If hyponatremia is related to a cerebral wasting syndrome (such as cerebral salt wasting), water restriction alone may not be the key treatment. In such cases, fluid and electrolyte replacement may be necessary.
- D) Hyponatremia during SIADH is typically hyposmolar, meaning that the serum osmolarity is decreased. Additionally, patients with SIADH typically have elevated urinary osmolarity, indicating an inappropriate retention of water by the kidneys.
- E) Urinary output measurement and blood tests, including serum sodium levels, are essential in the evaluation and management of hyponatremia. These tests help determine the severity of hyponatremia, guide the choice of treatment, and monitor the response to treatment.

It's important to note that the management of hyponatremia should be tailored to the individual patient's condition, and the underlying cause of hyponatremia should be identified and addressed accordingly.

**43. Which combination of the following is more likely to respond to an endoscopic third ventriculostomy for hydrocephalus?**

age < 1 month + postinfectious hydrocephalus + previously shunted patient

age 5 months + post hemorrhagic hydrocephalus + previously shunted patient

age 1 year + hydrocephalus associated with myelomeningocele + no previously shunted patient

age 2 years + aqueductal stenosis + previously shunted patient

age > 10 years + tectal tumor + no previously shunted patient

ChatGPT

The combination that is more likely to respond to an endoscopic third ventriculostomy for hydrocephalus is:

D) age 2 years + aqueductal stenosis + previously shunted patient

Explanation:

Endoscopic third ventriculostomy (ETV) is a surgical procedure used to treat hydrocephalus by creating an alternative pathway for the cerebrospinal fluid (CSF) to flow. The success of ETV depends on various factors, including the underlying cause of hydrocephalus.

Among the given options:

- A) Age < 1 month + postinfectious hydrocephalus + previously shunted patient: Infants younger than 1 month with postinfectious hydrocephalus may have complex hydrocephalus, and the success of ETV in this population is generally lower. Previous shunting also indicates a more complicated hydrocephalus case.
- B) Age 5 months + post hemorrhagic hydrocephalus + previously shunted patient: Post hemorrhagic hydrocephalus can be challenging to manage, and previous shunting indicates prior treatment failure. The success of ETV in this case may be lower.
- C) Age 1 year + hydrocephalus associated with myelomeningocele + no previously shunted patient: Hydrocephalus associated with myelomeningocele may have underlying anatomical abnormalities that can make ETV less effective. However, the absence of previous shunting may suggest a better response to ETV.
- D) Age 2 years + aqueductal stenosis + previously shunted patient: Aqueductal stenosis is a common cause of non-communicating hydrocephalus, and ETV can be an effective treatment option. The age of 2 years indicates a relatively older age group, which may have a better outcome with ETV compared to younger infants.
- E) Age > 10 years + tectal tumor + no previously shunted patient: Tectal tumors can cause hydrocephalus, but the treatment approach usually involves tumor resection rather than ETV. The age of >10 years may also influence the treatment decision, considering factors other than ETV.

Based on these considerations, option D (age 2 years + aqueductal stenosis + previously shunted patient) is more likely to respond to an endoscopic third ventriculostomy for hydrocephalus.

**44. Bobble-head doll sign is a clinical sign (2 right answers):**

associated with hydrocephalus and from obstructive third ventricular lesions

associated with hydrocephalus from fourth ventricle obstruction and medullary compression

it consists of oscillatory movements of the head and developmental delay

it consists of upward nystagmus in children

it affects mostly children from Asia

ChatGPT

The correct answers are:

A) Associated with hydrocephalus and from obstructive third ventricular lesions. B) Associated with hydrocephalus from fourth ventricle obstruction and medullary compression.

Explanation:

The bobble-head doll sign is a clinical sign associated with certain types of hydrocephalus and obstruction within the ventricular system. It is characterized by rhythmic, repetitive, and involuntary nodding or bobbing movements of the head. The movements are usually vertical and can be synchronous with the cardiac cycle.

Option A is correct because hydrocephalus can lead to increased intracranial pressure and obstruction within the ventricular system, including the third ventricle. Obstruction in this region can cause the characteristic bobble-head doll sign.

Option B is correct because obstruction at the level of the fourth ventricle, such as a mass or a blockage of the cerebrospinal fluid outflow pathways, can also result in hydrocephalus. The associated compression of the medulla can contribute to the bobble-head doll sign.

Option C is incorrect because the bobble-head doll sign is not specifically associated with developmental delay. It is primarily a motor sign related to abnormal head movements.

Option D is incorrect because upward nystagmus in children is not typically associated with the bobble-head doll sign.

Option E is incorrect because the bobble-head doll sign is not specific to children from any particular region or ethnicity. It can occur in individuals of different backgrounds who experience the underlying conditions causing hydrocephalus and ventricular obstruction.

**45. The caudal anterior limb of the internal capsule is supplied:**

By the middle cerebral artery

By the internal cerebral artery

By the recurrent artery of Heubner

By the posterior communicating artery

None of the above

ChatGPT

C) By the recurrent artery of Heubner

The caudal anterior limb of the internal capsule is primarily supplied by the recurrent artery of Heubner, also known as the medial lenticulostriate artery. This artery arises from the anterior cerebral artery (ACA) and provides blood supply to the basal ganglia, including the caudate nucleus and the anterior limb of the internal capsule.

Option A is incorrect because the middle cerebral artery (MCA) primarily supplies the lateral aspects of the cerebral hemispheres and is not the main source of blood supply to the caudal anterior limb of the internal capsule.

Option B is incorrect because there is no specific artery called the "internal cerebral artery." It may be a confusion with the term "internal carotid artery," which is a major artery supplying blood to the brain but does not directly supply the caudal anterior limb of the internal capsule.

Option D is incorrect because the posterior communicating artery is not directly involved in the blood supply to the caudal anterior limb of the internal capsule.

Option E is incorrect because there is a specific artery, the recurrent artery of Heubner, that supplies the caudal anterior limb of the internal capsule.

**46. The internal carotid artery enters the cranium via a canal formed by:**

The shenoid bone

The occipital bone

The sphenoid and temporal bone

The temporal bone

The temporal and occipital bone

ChatGPT

A) The sphenoid bone

The internal carotid artery enters the cranium through the carotid canal, which is a passageway formed by the sphenoid bone. The carotid canal is located in the petrous part of the temporal bone and extends from the middle cranial fossa to the base of the skull.

Option B is incorrect because the occipital bone does not contribute to the formation of the carotid canal.

Option C is incorrect because the carotid canal is primarily formed by the sphenoid bone, with minimal contribution from the temporal bone.

Option D is incorrect because the temporal bone is involved in the formation of other structures, such as the internal acoustic meatus, but not the carotid canal.

Option E is incorrect because the occipital bone is not part of the carotid canal, and the temporal bone's contribution to the carotid canal is minimal.

**47. A 65 year old male patient presents with progressive weakness and numbness in the right lower extremity. Neurological examination reveals hyperreflexia and positive Babinski sign on the right side. An MRI scan shows a large intradural extramedullary tumor located at the level of the thoracic spinal cord. What is the most likely diagnosis for this patient?**

Meningioma

Glioblastoma

Schwannoma

Ependymoma

Medulloblastoma

ChatGPT

C) Schwannoma

Based on the clinical presentation and MRI findings described, the most likely diagnosis for this patient is a schwannoma. Schwannomas are typically benign tumors that arise from Schwann cells, which are the cells that wrap around and insulate nerve fibers. They commonly occur in the intradural extramedullary space, and their location along the spinal cord can cause compression and resulting neurological symptoms.

Meningiomas (Option A) are another type of intradural extramedullary tumor, but they arise from the meninges, the protective membranes covering the brain and spinal cord. They can occur at various locations along the spinal cord.

Glioblastoma (Option B) and medulloblastoma (Option E) are types of intracranial tumors and are less likely to present as an intradural extramedullary tumor at the level of the thoracic spinal cord.

Ependymomas (Option D) are intramedullary tumors that arise from ependymal cells lining the central canal of the spinal cord. They are less likely to present as an extramedullary tumor.

Given the clinical presentation, imaging findings, and the common location of schwannomas along the spinal cord, it is the most likely diagnosis in this case.

**48. A 45-year-old patient presents with severe headaches, visual disturbances, and bitemporal hemianopia. Magnetic resonance imaging (MRI) reveals a large pituitary tumor compressing the optic chiasm. What is the most appropriate treatment for this patient?**

Craniotomy and microsurgical resection of the tumor

Gamma-knife radiosurgery

Transphenoidal endoscopic resection of the tumor

Medical managmenet with dopamine agonists

Observation and follow-up with serial imaging

ChatGPT

(Note: The answer choices are based on common treatment options for pituitary tumors, and the most appropriate choice may vary depending on the specific clinical scenario.)

**49. Describe the clinical features and diagnostic workup of a patient presenting with symptoms of hydrocephalus. What are the appropriate treatment options for this condition?**

ChatGPT

Hydrocephalus is a condition characterized by an abnormal accumulation of cerebrospinal fluid (CSF) within the ventricles of the brain, leading to increased intracranial pressure. The clinical features and diagnostic workup of a patient presenting with symptoms of hydrocephalus, as well as the appropriate treatment options, are as follows:

Clinical Features:

1. Infants and young children:
   - Rapid head growth
   - Bulging fontanelle (soft spot)
   - Separation and widening of skull sutures
   - Irritability and fussiness
   - Poor feeding
   - Vomiting
   - Downward deviation of the eyes (sunsetting sign)
   - Seizures
2. Older children and adults:
   - Headache
   - Nausea and vomiting
   - Blurred or double vision
   - Changes in gait and coordination
   - Difficulty in controlling bladder and bowel movements
   - Cognitive and memory problems
   - Personality changes
   - Drowsiness or lethargy

Diagnostic Workup:

1. Neurological examination: Assessing neurological signs, such as abnormal reflexes, muscle weakness, and changes in sensory function.
2. Imaging studies:
   - Cranial ultrasound (in infants) or CT scan/MRI of the brain: To visualize the ventricles and identify the presence of hydrocephalus and any underlying causes.
   - Cine MRI or CSF flow study: To evaluate CSF flow dynamics and identify potential obstructions or abnormalities.
3. Lumbar puncture: Measurement of CSF pressure and analysis of its composition.

Treatment Options:

1. Shunt Placement: The most common treatment for hydrocephalus is the surgical placement of a shunt system. A shunt consists of a tube that diverts excess CSF from the ventricles to another body cavity, such as the peritoneal cavity or the right atrium of the heart. This helps to regulate the CSF flow and relieve intracranial pressure.
2. Endoscopic Third Ventriculostomy (ETV): This is an alternative surgical procedure in which a small hole is made in the floor of the third ventricle to allow CSF to bypass any obstruction and flow freely within the subarachnoid space.
3. Treatment of the underlying cause: If hydrocephalus is secondary to another condition, such as a tumor or infection, treating the underlying cause is essential. This may involve surgery, radiation therapy, chemotherapy, or antimicrobial therapy.
4. Medications: In some cases, medications may be prescribed to reduce CSF production or improve CSF absorption.
5. Regular follow-up and monitoring: Patients with hydrocephalus often require long-term monitoring and adjustments to their treatment plan to ensure optimal management and minimize complications.

The specific treatment approach for hydrocephalus depends on factors such as the underlying cause, the age of the patient, the severity of symptoms, and individual patient considerations. The choice of treatment should be made in consultation with a neurosurgeon or neurologist experienced in managing hydrocephalus.

**50. Which structure serves as a landmark to identify the lateral ventricle during neurosurgical procedure?**

Cingulate gyrus

Corpus callosum

Thalamus

Hippocampus

ChatGPT

B) Corpus callosum

The corpus callosum serves as a landmark to identify the lateral ventricle during neurosurgical procedures. It is a large bundle of nerve fibers that connects the two cerebral hemispheres. The lateral ventricles lie within the cerebral hemispheres, and the corpus callosum runs above them, separating the lateral ventricles from each other. Surgeons often use the corpus callosum as a guide to locate and navigate the lateral ventricles during procedures such as ventriculostomy or tumor resection.

Note for the reader:

*Questions 7, 8, 11, 14, 15, 24, 33, 34 of the exam were not submitted to ChatGPT since they contained images to interpret or an institutional protocol to know.*
